# Pupil Mimicry is the Result of Brightness Perception of the Iris and Pupil

**DOI:** 10.5334/joc.34

**Published:** 2018-06-19

**Authors:** Madou Derksen, Juliette van Alphen, Sander Schaap, Sebastiaan Mathot, Marnix Naber

**Affiliations:** 1 Experimental Psychology, Helmholtz Institute, Utrecht University, NL; 2 Department of Experimental Psychology, University of Groningen, NL

**Keywords:** Pupil, Mimicry, Dilation, Visual Attention

## Abstract

Recent scientific investigations suggest that people automatically mimic each other’s pupil sizes during interaction. However, instead of being a social mimicry effect, it could also be the result of brightness perception. When observers look at individuals with dilated pupils, little of the brighter iris is visible, leading to the perception of a relatively low-illuminated eye region. In the current study we tested whether pupil mimicry remains present when pupils and irises are equalized for luminance values across pupil sizes. We tested several stimulus sets, including faces with static pupils that varied in size across images and dynamic pupils that changed in size over time in videos. Results showed that for traditional, not-luminance-equalized videos, participants’ pupil sizes adapted to the observed pupils, showing a pattern that is roughly in line with pupil mimicry. However, no such pupil response in line with mimicry was seen for static images (regardless of whether they were equalized for luminance) nor for luminance-equalized videos. These findings suggest that only salient, dynamic stimuli attract enough attention to the luminance in the eye region to evoke a pupillary response. However, although such responses suggest pupil mimicry, the underlying factor is the change in brightness within the eye as a function of pupil size rather than social mimicry.

## 1. Introduction

A person’s tendency to mimic others’ behavior is surprisingly strong. Mimicry is a dominant factor in driving behavior, independent of the circumstances, whether it is in a social or non-social situation (Chartrand & Bargh, 1999; Dijksterhuis & Bargh, 2001; Lakin, Jefferis, Cheng, & Chartrand, 2003; Naber, Vaziri Pashkam, & Nakayama, 2013). Perhaps the most remarkable demonstration of mimicry is that of pupil mimicry (Fawcett, Arslan, Falck-Ytter, Roeyers, & Gredebäck, 2017; Fawcett, Wesevich, & Gredebäck, 2016; Harrison, Gray, & Critchley, 2009; Harrison, Singer, Rotshtein, Dolan, & Critchley, 2006; Kret & De Dreu, 2017; Kret, Fischer, & De Dreu, 2015; Kret, Tomonaga, & Matsuzawa, 2014). Pupil mimicry, often referred to as pupil contagion, is the phenomenon that an observer’s own pupil size is adjusted towards the size of the eye’s pupil of an observed individual. This effect has been reported to occur in infants (Fawcett et al., 2017; Fawcett et al., 2016) and adults (Harrison et al., 2009; Harrison et al., 2006; Kret & De Dreu, 2017; Kret et al., 2015; Kret et al., 2014). Even chimpanzees are subject to pupil mimicry according to recent findings (Kret et al., 2014). Similar to the belladonna hypothesis, suggesting that faces with dilated pupils are rated as more attractive (Hess, 1965), pupil mimicry seems to be driven by a social mechanism. Note however that the suggested link between attractiveness of faces and pupil size was not confirmed in several studies (Demos, Kelley, Ryan, Davis, & Whalen, 2008; Janisse, 1973; Laeng & Falkenberg, 2007). Nonetheless, it is generally assumed that pupil mimicry is driven by a social mechanism.

The finding that humans automatically mimic each other’s pupil sizes is surprising, because pupil size is an inconspicuous signal. Observers are typically unaware of changes in others’ pupil size (Naber, Stoll, Einhäuser, & Carter, 2013). Nevertheless, several studies on pupil mimicry have circumvented the issue of conspicuity by presenting only regions of the face showing the eyes (i.e., eye slits) rather than full faces (Kret & De Dreu, 2017; Kret et al., 2015; Kret et al., 2014). These studies also added a dynamic component to the pupil by having it increase or decrease in size over time. By showing merely the eyes with sudden changes in pupil size, attention is very likely drawn specifically to the pupils. Because the visibility of the observed behavior mediates the strength of mimicry (Naber, Vaziri Pashkam, et al., 2013), any additional focus on observed pupil sizes strengthens the possibility of pupil mimicry.

The important role of the focus of attention opens up the possibility that pupil mimicry may not be the result of the automatic perception and mimicry of pupil size. Instead, a potential confound of eye brightness may explain the adaptation of pupil sizes when observing changes in pupil size. For example, when observers focus on a large, dilated pupil, this results in an increase in the dark pupil area and decrease in the bright iris area as compared to when the pupil is constricted. In other words, a person with a large pupil displays a darker, less illuminating eye as compared to a person with a small pupil.

**Figure 1:**
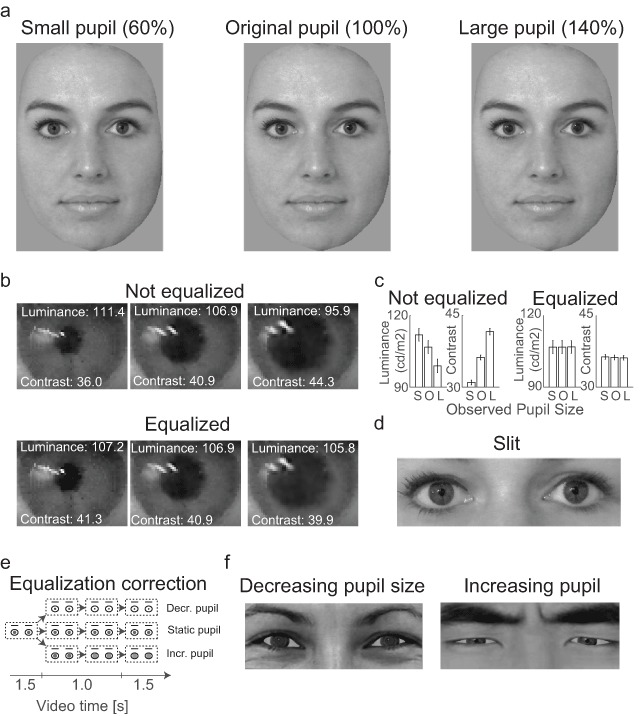
Full face stimuli with different pupil sizes were presented in the first block of trials **(a)**. Example of eye regions before and after luminance/contrast equalization **(b)**. The eyes’ pixel values were equalized for luminance and contrast **(c)** such that eyes with large pupils got brighter with less contrast while small pupils got darker with more contrast (b). Slits of the eye region of the faces were shown in the second and third block of trials **(d)**. Schematic overview of the procedure of luminance equalization for the video stimuli in experiment 2 **(e)**. Snapshots of a happy, European, female face with dilated pupils (left) and an angry, Japanese, male face with constricted pupils (right) in which the brightness of the pupil was corrected to ensure equal luminance across video frames as a function of pupil size **(f)**. These images were adapted with permission from Kret et al. (2015).

Although it is well known that perceived brightness and contrast affect pupil size (Mathôt & Van der Stigchel, 2015; Binda, Pereverzeva, & Murray, 2013b; Laeng & Endestad, 2012; Naber et al., 2011; Naber & Nakayama, 2013), it is tempting to denote their effects as negligible in pupil mimicry experiments. Differences in luminance across small, original, and large pupil conditions within the eye regions of a stimulus are relatively small with respect to the global variations in luminance across the entire stimulus surface (Figure 1). Thus, pupil responses could be, in theory, driven mainly by global rather than local luminance and contrast levels, independent of the focus of attention. However, recent discoveries suggest that the focus of attention on the brightness of local objects strongly determines pupil responses (Binda, Pereverzeva, & Murray, 2013a; Mathôt, Van der Linden, Grainger, & Vitu, 2013; Naber, Alvarez, & Nakayama, 2013). More specifically, several studies have discovered that if a dark and bright stimulus are shown at the same time and at equal distance from fixation in the periphery, and if only the dark stimulus is covertly attended (i.e., paying attention to but not gazing at the dark stimulus), then the observer’s pupils dilate as if mainly the dark stimulus is observed. It can be concluded from these studies that also local luminance within the focus of attention drives pupillary responses. Because eyes are conspicuous objects, it is not unlikely that attention is often drawn covertly or overtly to the eye’s local luminance features during pupil-mimicry experiments. While previous pupil mimicry studies have controlled for global luminance (e.g., Fawcett, Arslan, Falck-Ytter, Roeyers, & Gredebäck, 2017; Kret, Fischer, & De Dreu, 2015), these studies have not controlled for local luminance in the eye regions.

Here we investigated to what degree the pupil mimicry effect depends on the local luminance variations across pupil size conditions. In two separate experiments we explored whether the pattern of pupil mimicry can be explained by the conspicuity and brightness levels of the eye region. We used a variety of stimulus conditions to test this: (1) traditional, uncorrected images of slits of the eyes with three different pupil-size conditions per image, (2) corrected images of slits of the eyes with equalized luminance and contrast within the eye regions across pupil-size conditions, (3) full face images expressing a variety of emotions with equalized local luminance and contrast, (4) uncorrected videos of slits of the eyes in which the pupil dynamically becomes bigger of smaller, and (5) luminance-equalized videos of the eyes in which the pupil becomes brighter or darker as it decreases or increases in size, respectively.

## 2. Methods

### 2.1. Observers

Sixty observers (42 female, *M* = 21.6, *STD* = 2.23) participated in experiment 1 and a new set of sixty-six observers (51 female, *M* = 21.2, *STD* = 1.31) in experiment 2. All observers had normal or corrected-to-normal vision. The observers were approached personally or with flyers at Utrecht University campus. Prior to the experiments each observer signed an informed consent form. The observers were unaware of the purpose of the experiments. Observers were only told in experiment 1 that the purpose of the study was to investigate how people looked at faces using an eye tracker. In experiment 2 the observers were told that the purpose of the study was to investigate the effects of trust on gaze behaviour. The recording of pupil size was not mentioned. Before participating in the experiments observers were offered the choice between study credit or a monetary compensation of 6 euros for participation.

### 2.2. Apparatus

Stimuli were shown on an LED Asus ROG swift pg278q (AsusTek Computer Inc., Taipei, Taiwan) computer monitor (68.6 cm diagonal; 16:9 ratio) with a resolution of 2550 × 1440 pixels, a refresh rate of 60 Hz, and maximum luminance of 344 cd/m^2^ with ultra-low motion blur (ULMB) turned off. The screen was linearized (gamma = 1) such that computer input pixel values were linearly related to the LED output brightness (i.e., a two-fold increase in pixel value corresponded to a two-fold increase in brightness). Stimuli were presented with MatLab 2014b (Mathworks, Natick, MA, USA) using the PsychToolbox extension (Brainard, 1997; Pelli, 1997). Observers sat in a chair with their head in a chin- and forehead-rest at a fixed viewing distance of 53cm from the monitor. Pupil size and gaze of the right eye was recorded with an Eyelink 1000 eye-tracker (SR Research, Ontario, Canada) placed 40 cm in front of the observer right under the monitor. Eye-tracker calibration consisted of a thirteen-point grid. The experiment was conducted in a darkened room without ambient light.

### 2.3. Stimuli

#### 2.3.1. Images for experiment 1

The stimuli of experiment 1 were chosen from a database that consisted of 80 high-resolution pictures (2448 × 3264 pixels) of the faces of European adults (61 female) with blue, green, or grey irises. The faces expressed different emotions (neutral, happy, sad, angry; 20 images per emotion). The faces were cropped such that only facial skin and no ears, neck, or hair were visible (see Figure 1a). The surfaces of the cropped faces were equalized in size such that all face stimuli consisted of 50002 ± 36 pixels (*M* = 922 ± 37 by 663 ± 24 pixels). Next, the image parts displaying the eyes of the faces were cut out (eye white, iris, and pupil) and saved for later pupil size manipulations. All remaining 80 eye-less faces were equalized in luminance and contrast by using a histogram equalization approach in which the histograms of pixel values of each image was adjusted to match with the average histogram of all images. The average luminance and contrast (standard deviation of all pixel values) of the eye-less faces was 210 cd/m^2^ ± 0.12 and 37 cd/m^2^ ± 0.11, respectively.

In addition to the faces with original pupil sizes, a large pupil size condition (i.e., 140% of the original pupil size) and a small pupil condition (i.e., 60% of the original pupil size) was created. Pupil size was manipulated in a way that the naturalistic appearance of the pupils was not affected. The pupil conditions were created by (i) manually selecting the pupil from the previously cut-out eye parts of face images, by (ii) resizing the selected pupil area using a biharmonic spline 2D interpolation method (i.e., v4). Next, the eyes were equalized for luminance and contrast by adjusting the pixel values of the eye regions for the faces with small and large pupils such that it matched the average pixel value of the original pupil condition (for before and after equalization, see Figure 1b). Before equalization the average luminance was higher for eye regions with small pupils and lower for large pupils as compared to original pupils (Figure 1c). On the contrary, the contrast of the eye regions was smaller for small pupils while larger for large pupils as compared to the original pupils. The average difference in luminance and contrast between the small and original pupil condition was 5.13 cd/m^2^ ± 2.12 and 5.26 cd/m^2^ ± 2.17, respectively. The difference in luminance and contrast between the original and large pupil condition was 7.91 cd/m^2^ ± 4.21 and 5.42 cd/m^2^ ± 3.32, respectively. After equalization the average difference in luminance and contrast was small with 0.01 cd/m^2^ ± 0.06 and 0.12 cd/m^2^ ± 0.31 between the small and original pupil condition, and 0.02 cd/m^2^ ± 0.12 and 0.06 cd/m^2^ ± 0.76 between the original and large pupil condition.

In addition to the creation of full face stimuli, additional stimuli were created to replicate a previous study in which only slits of the faces merely containing the eyes were presented (Kret et al., 2015; Kret et al., 2014). Only the faces with a neutral expression were used for the slit stimuli (i.e., 20 pictures). See Figure 1d for examples of slit face stimuli.

Prior to the experiment, the emotions of the faces were validated by having seven participants classify the pictures on emotional expression. These participants did not partake in the main experiment. Table 1 shows a confusion matrix of the average recognition scores across participants in percentages, with rows indicating the presented emotions (ground truth) and columns indicating the observed emotions as reported by the participants.

**Table 1:** Confusion matrix with the average and standard error of percentages of the observed emotional expressions in experiment 1 across participants (subjective; columns) per shown emotional expression (objective ground truth; rows).

Confusion matrix %	Happy	Neutral	Angry	Sad

Happy	97% ± 1	1% ± 0	1% ± 1	1% ± 1
Neutral	2% ± 1	98% ± 1	0% ± 0	0% ± 1
Angry	0% ± 0	4% ± 1	94% ± 1	2% ± 1
Sad	0% ± 0	4% ± 2	4% ± 2	92% ± 2

#### 2.3.2. Videos for experiment 2

The stimuli of the second experiment consisted of videos of slits of the eyes with pupils that either decreased, remained static, or increased in size. The videos were 4 seconds long during which the pupil remained static for the first 1.5 seconds, followed by either a change or no change in pupil size for approximately 1 second, and then a stabilization of pupil size for another 1.5 seconds. The original, not-luminance-equalized videos were taken from the stimulus set developed by Kret et al. (2015), based on images of two other stimulus sets (Matsumoto & Ekman, 1989; Van Der Schalk, Hawk, Fischer, & Doosje, 2011), that included 4 female and 4 male European and 4 female and 4 male Japanese characters with either happy or angry expressions. The same video set was used to create luminance-equalized videos. The pupil area was selected automatically by a threshold rule (all pixels with a gray value below 20). The baseline pupil at the first 1.5 seconds of each video was increased in luminance by adding 20 gray values. Next, the luminance of the pupil was dynamically varied as a function of pupil size across the frames, ensuring that the luminance within the iris region (i.e., average luminance of all pixel values belonging to the iris and pupil) remained unchanged and thus equalized over time (Figure 1e–f).

### 2.4. Procedure

The first experiment consisted of three blocks. The full face and not the slit stimuli were shown in the first block. If the slit stimuli had been presented before or intermixed with the full face stimuli, then the participants would have been primed about the relevance of the eyes in the face stimuli and could have artificially drawn attention towards the eye regions. We felt that it was relevant to investigate whether pupil contagion is a general phenomenon in the sense that the face stimuli should be able to evoke pupil mimicry without observers paying special attention to the eye regions and pupils, just as during real-life interaction. The full face-stimulus block consisted of a total of 3 (pupil size conditions) times 4 (emotions) times 20 (faces) is 240 trials and took approximately 15 minutes to complete. A fixation point was shown right between the eyes of the faces, and participants were instructed to fixate on this point and not to make eye-movements. The fixation point was also shown during face presentation to ensure that pupil size was not affected by changing gaze input and saccades (Knapen et al., 2016).

The slits of the eyes of neutral faces without and with luminance and contrast adaptations of the eye regions were shown in the second and third block. These blocks consisted of a total of 3 times 20 is 60 trials and took approximately 4 minutes to complete. In contrast to the face stimuli, but in line with the previous studies that presented slits of the eyes, participants were allowed to make eye-movements during presentation of the slits. Therefore the fixation point was removed when a slit stimulus was shown.

Participants were instructed to pay close attention to the stimuli. Except for inspecting the stimuli, participants performed no tasks. Each stimulus was shown for 3 seconds and preceded by a blank screen that was shown for an interval randomly chosen from 0.5 to 1.5 seconds to ensure that the onset of the stimuli were unpredictable. A fixation point was shown before stimulus presentation in all blocks.

The procedure of the second experiment was equal to the first block of experiment 1, except for the amount of trials, inter-stimulus-interval, and instructions. The video stimuli consisted of a total of 3 (pupil-size conditions) times 2 (emotions) times 8 (faces) times 2 (groups) times 2 (luminance equalization) is 192 trials. Following the design of Kret et al. (2015), a trial consisted of a 4s inter-stimulus-interval period in which a phase-scrambled image of the first frame of the upcoming video was presented, and a 4s presentation period in which the video of the face of a person was shown. A fixation cross was presented between the eyes 0.5s before video onset. The participants were instructed to think about to what degree they would trust and invest money in a company run by the depicted person. Each video was followed by the question whether they would or would not invest €5 in the depicted person. The outcomes of this investment game were not analyzed because it falls outside the scope of the current manuscript.

### 2.5. Statistics

Note that the Eyelink pupil tracking system outputs pupil size in arbitrary units rather than absolute pupil diameter in millimetres. To allow comparisons across participants, pupil size was converted to baseline-corrected z-scores by subtracting the pupil size at stimulus onset (experiment 1) or pupil change onset (experiment 2) from the time series of pupil size per trial (i.e., baseline correction), and by then dividing pupil size by the standard deviation of pupil size across all time points and trials (i.e., *z*-scoring). For the data of experiment 2 an additional correction was applied. The pupil constricts and then dilates back to baseline in response to the onset of motion (Barbur, Harlow, & Sahraie, 1992). To remove this effect, each pupil response to an observed increasing or decreasing pupil was subtracted (1) by the average normalized pupil responses of the increasing and decreasing pupil condition per group and luminance equalization condition and (2) by adding the average normalized pupil responses of the static pupil condition. This results in a relative change in pupil size that incorporates the general trend of the pupil responses over time but does not incorporate the confounding effect of motion.

After normalization, two analyses were performed on the pupil size measurements. First, pupil size was calculated as a function of time after stimulus onset (time series) per observed pupil size (and emotion condition for data from block 1) to provide an overview of the pattern of pupil response across conditions. Second, average pupil size was calculated between 1 and 3 seconds in the time series after image onset for static images and between 1 and 2.5 seconds after pupil change onset for videos (i.e., around peak pupil sensitivity to stimulus content, incorporating a typical 1s response delay). A repeated measures analysis of variance (ANOVA) was performed with observed pupil size (i.e., what the participant saw) and emotion for block 1 in experiment 1, and observed pupil size, emotion, group, and luminance equalization for experiment 2 as independent variable(s), and average observer’s pupil size as dependent variable. For experiment 1, a third analysis was performed: pupil size was calculated per stimulus character (i.e., per European adult) to take into account variations caused by differences in image statistics across faces. These variations were then removed by subtracting the time series of the original pupil size condition from the small and large pupil size conditions per stimulus character. By computing only the relative pupil response (i.e., relative to the original pupil size condition), this data correction ensured that statistical outcomes were invariant to the effects of stimulus character on pupil responses. This third analysis step was not necessary for the data of experiment 2 because here relative data was already computed by the additional normalization correction step (see previous paragraph). We also created a linear mixed-effects model containing those factors that Kret et al. (2015) found to be important. Besides using the same model structure and variable scaling (see their manuscript for details), we added the factor luminance equalization as a fixed effect, to test whether the original results of Kret et al. (2015) are modulated (i.e., interact with) by luminance modifications as a function of pupil size. In addition to the conventional ANOVA tests and linear mixed-effects model, Bayesian statistics were performed with the program JASP (Love et al., 2015).

The percentage of viewing time inside and outside the eye regions was calculated for the trials with slit stimuli because these trials displayed no fixation during stimulus presentation. Fixations belonged to the inside eye region when gaze coordinates felt on top of the sclera (including eye lashes), iris, or pupil areas, covering 14% of the total slit images.

Statistical outcomes were calculated in JASP each time an additional 20 participants were recruited. Bayesian statistics allowed it to determine when enough evidence was collected in favour or against the null-hypothesis (Dienes, 2014; Wagenmakers, 2007). Instead of performing a prior power analysis, we applied the following stopping rule: a minimum of 60–70 participants because previous pupil mimicry studies had recruited similar or less amounts of participants (Fawcett et al., 2016; Harrison et al., 2009; Harrison et al., 2006; Kret et al., 2015; Kret et al., 2014), and all Bayes factors for the main-effects of observed pupil size should indicated enough evidence (i.e., moderate: BF > 3 or BF < 1/3) in favour of the h0 or h1 hypothesis after the recruitment of 60–70 participants.

## 3. Results

### 3.1. Slit of the eye regions, not controlled for luminance and contrast

**Figure 2:**
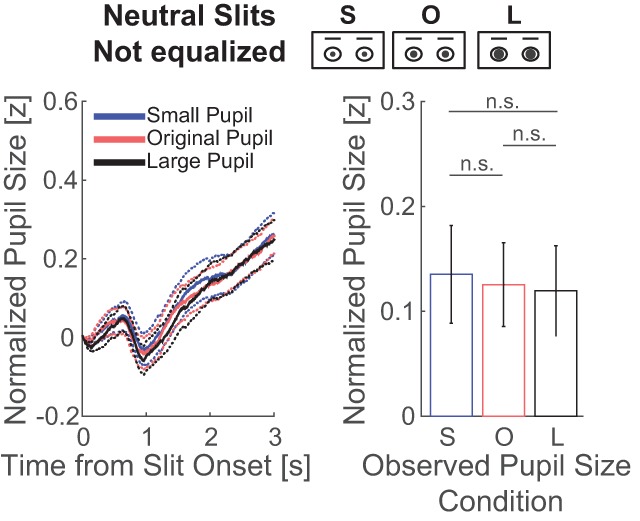
The left plot shows the average change in pupil size across observers as a function of time from slit onset (solid lines) per observed pupil-size condition (small pupil: blue; original pupil: red; large pupil: black) in experiment 1. The displayed eye regions in the slits were not equalized in luminance and contrast. The dotted lines around the solid lines indicate standard errors of the mean. The right plot shows the average and standard error of the change in pupil size across observers, after averaging between 1 and 3 seconds from slit onset, per observed pupil-size condition (small: S; original: O; large: L; n.s.: not significant).

We first analyzed whether the pupil-contagion effect was present when no adaptations were made to local stimulus luminance and contrast in the eye regions. As shown in Figure 2, pupil size as a function of time from slit onset showed no differences across the observed pupil-size conditions. The main effect of observed pupil size on average observers’ pupil size between 1–3 seconds after slit onset was not significant with strong evidence *against* a pupil-contagion effect (*F*(2,118) = 0.06, *p* = 0.940, η_p_^2^ = 0.001, BF10 = 0.059; for mean and confidence intervals of differences between pupil size conditions, see Table 2; for pupil traces per observer, see Figure S1a in the Supplemental Material available online). A non-significant main effect of pupil size was reconfirmed with an ANOVA on the data corrected for effects of stimulus characteristics (for details, see Methods) with moderate evidence *against* a pupil-contagion effect (*F*(1,59) = 0.11, *p* = 0.746, η_p_^2^ = 0.002, BF10 = 0.204). The total view time of the eye regions was 30 ± 10% and this number differed across pupil size conditions (small pupil: 29.1%, original pupil: 29.7%, large pupil: 30.2%; *F*(2,118) = 3.87, *p* = 0.024). This effect was however small (η_p_^2^ = 0.062) and inconclusive according to Bayesian statistics (BF10 = 1.51).

**Table 2:** Means, confidence intervals, and post-hoc t-test comparisons across pupil size conditions per block in experiment 1 (Blo.).

Blo.	Compared conditions		Mean difference	Confidence interval	*t*-value	*p*-value	Cohen’s D	BF

1	Small	Original	0.01	–0.08/0.10	0.22	0.823	0.233	0.145
	Original	Large	0.02	–0.08/0.09	0.13	0.894	0.235	0.142
	Small	Large	0.01	–0.08/0.11	0.33	0.745	0.188	0.149

2	Small	Original	–0.10	–0.17/–0.01	2.35	**0.022**	**0.438**	**1.778**
	Original	Large	0.06	–0.02/0.15	1.46	0.149	0.132	0.386
	Small	Large	–0.03	–0.13/0.06	0.70	0.490	0.195	0.178

3	Small	Original	0.02	–0.02/0.06	0.96	0.339	0.068	0.220
	Original	Large	–0.02	–0.06/0.02	1.08	0.287	0.079	0.244
	Small	Large	–0.00	–0.04/0.03	0.15	0.882	0.010	0.143

The lack of an effect of observed pupil size was unexpected as previous studies found evidence for pupil contagion with similar stimuli (Kret et al., 2014). A potential explanation for these null results is that differences in luminance contrast within the eye regions suppressed the pupil-contagion effect. Contrast was higher in eye regions with large pupils as compared to small pupils (see Methods), potentially evoking more pupil constriction and therewith counteracting the pupil contagion effect. The following analysis assesses pupil responses to stimuli that were equalized for luminance and contrast within the eye regions.

### 3.2. Slit of the eye regions, controlled for luminance and contrast

**Figure 3:**
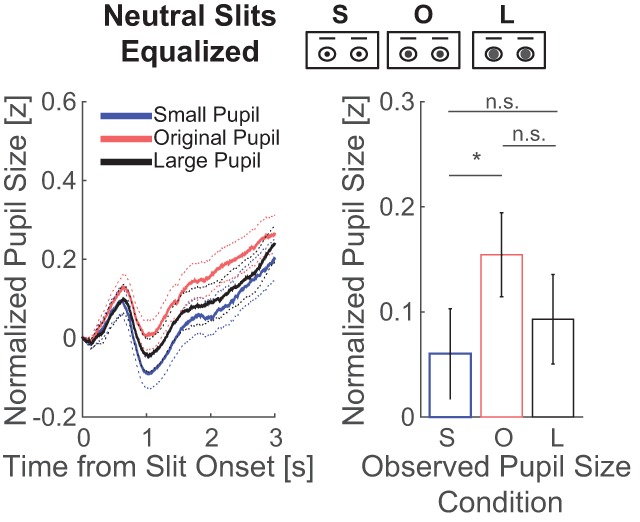
The left plots shows the average change in pupil size across observers as a function of time from slit onset per observed pupil size condition in experiment 1. The eye regions presented in the slits were equalized in luminance and contrast. The right plot shows the average and standard error of the change in pupil size across observers, after averaging between 1 and 3 seconds from slit onset, per observed pupil-size condition (small: S; original: O; large: L; n.s.: not significant; * < 0.05).

The second analysis centered around the question whether the pupil-contagion effect is present when displaying slits of the eyes that have equal luminance and contrast across the pupil-size conditions. Figure 3 displays the pattern of pupil responses across observed pupil-size conditions. The time series, however, showed a nonlinear pattern in which the original, intermediate pupil-size condition caused slightly more pupil dilation than small and large pupil size conditions. However, statistics indicated neither a significant main effect of observed pupil size on uncorrected pupil size of the observers (*F*(2,118) = 2.45, *p* = 0.091, η_p_^2^ = 0.040, BF = 0.471; for mean and confidence intervals of differences between pupil size conditions, see Table 2; for pupil traces per observer, see Figure S1b in the Supplemental Material available online), nor a main effect on the relative, corrected pupil size with moderate evidence in favor of the null hypothesis of no pupil contagion effect (*F*(1,59) = 0.48, *p* = 0.490, η_p_^2^ = 0.008, BF = 0.237). The total view time of the eye regions was 29 ± 9% and did not differ across pupil size conditions (small pupil: 31.0%, original pupil: 30.8%, large pupil: 30.5%; *F*(2,118) = 1.14, *p* = 0.322; η_p_^2^ = 0.019; BF = 0.165).

Again, data showed no evidence in favor of the existence of pupil contagion, despite controls for luminance and contrast. One reason for this could be that the pupil-contagion effect is present when the manipulated pupil sizes are observed in faces that express a sad emotion but not in faces with a neutral or other emotional expression (Harrison et al., 2006). The next analysis will explore the factor of emotional expression.

### 3.3. Full faces with emotional expressions, controlled for luminance and contrast

**Figure 4:**
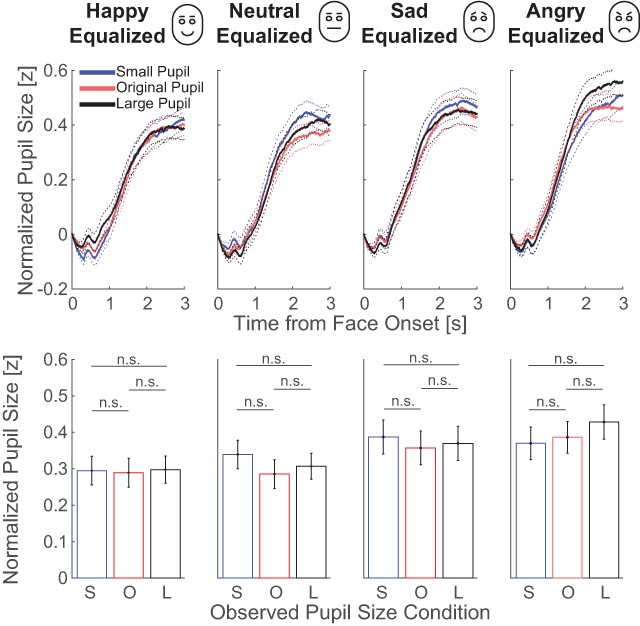
The top row shows the average change in pupil size across observers as a function of time from face onset per emotional expression condition in experiment 1. The dotted lines around the solid lines indicate standard errors of the mean. The bottom row shows the average and standard error of the change in pupil size across observers, after averaging between 1 and 3 seconds from face onset, per observed pupil-size condition (small: S; original: O; large: L; n.s.: not significant).

The analysis of this part focused on the question whether the pupil-contagion effect only occurs when sad faces were presented. Similar to the previous stimulus set, eye regions were equalized for luminance and contrast across observed pupil size conditions. Figure 4 displays the pupil responses to the onset of the face images per observed-pupil-size condition and per emotion condition (for pupil traces per observer, see Figure S2 in the Supplemental Material available online). These plots showed no pattern in line with the pupil-contagion effect. Statistics confirmed no significant main effect of observed pupil size with strong evidence *against* a pupil-contagion effect (*F*(2,118) = 0.73, *p* = 0.484, η_p_^2^ = 0.012; BF10 = 0.029; for mean and confidence intervals of differences between pupil size conditions, see Table 2). The degree of pupil dilation did differ between emotion conditions (compare dilation patterns across subplots in Figure 4), with the negative emotion conditions (sad and anger) evoking larger pupils than the positive (happy) and neutral (*F*(3,177) = 7.93, *p* < 0.001, η_p_^2^ = 0.118; BF10 = 594.998; see Supplementary Table S1 for post-hoc comparisons). The absence of an interaction between emotion and pupil size indicated that the pupil-contagion effect was not modulated by emotion and thus not present for sad emotions or any other subset of emotions (*F*(6,354) = 0.60, *p* = 0.733, η_p_^2^ = 0.010; BF10 = 0.111). A non-significant main effect of pupil size (*F*(1,59) = 0.02, *p* = 0.885, η_p_^2^ < 0.001; BF10 = 0.100) and a lacking interaction with emotion (*F*(3,177) = 1.10, *p* = 0.350, η_p_^2^ = 0.018; BF10 < 0.001) was reconfirmed with the data corrected for effects of stimulus characteristics with strong evidence against relevant effects. In sum, the pupil-contagion effect was not present in any of the emotion conditions.

To summarize the results of experiment 1, a pattern of pupil mimicry was not found for images of faces with variable pupil sizes across images. This suggests that it is challenging to replicate pupil-mimicry effects using images with static pupils. However, recent studies used dynamic rather than static stimuli, consisting of videos in which the pupil transiently dilated or constricted (Kret & De Dreu, 2017; Kret et al., 2015; Kret et al., 2014). One can imagine that such a transient response draws attention to the pupil, therewith strengthening the pupil mimicry effect. In the following experiment we used dynamic video of changing pupil sizes, either corrected or uncorrected for luminance within the eye regions, to investigate whether pupil mimicry holds under variable image-statistics conditions.

### 3.4. Videos of slits of the eyes with dynamically changing pupil sizes

**Figure 5:**
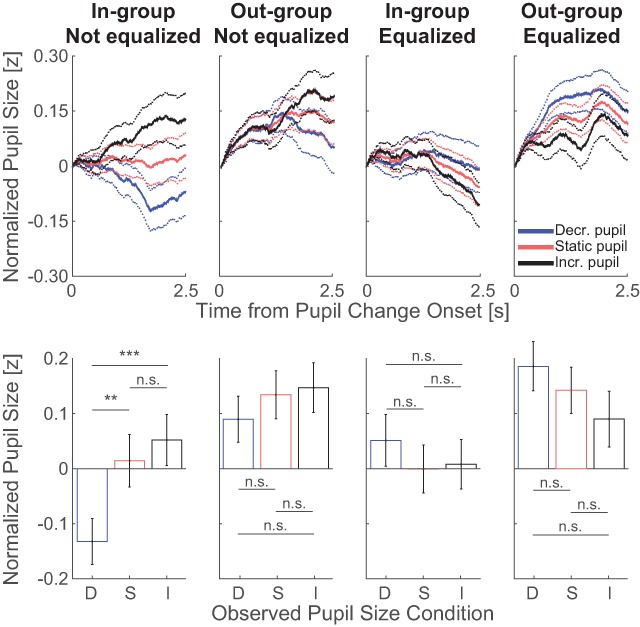
Top row shows average change in pupil size across observers as a function of time from observed pupil change onset per pupil condition in experiment 2. Bottom row shows average and standard error of change in pupil size across observers, after averaging between 1.0 and 2.5s from observed pupil change onset, per observed pupil-size condition (decreasing: D; static: S; increasing: I; n.s.: not significant; ** < 0.01, *** < 0.001). Data were split per luminance-not-equalized (1^st^ and 2^nd^ columns) and luminance-equalized (3^rd^ and 4^th^ columns) pupils, and in-group (1^st^ and 3^rd^) and out-group faces (2^nd^ and 4^th^).

Experiment 2 centered on the question whether the pupil-contagion effect is present when displaying slits of the eyes of European (in-group) and Japanese (out-group) faces with pupils that dynamically change in size, and were or were not equalized for luminance as a function of changing pupil size. Figure 5 displays the pattern of pupil responses per observed pupil size (gray values) and per condition (subplots). A four-way ANOVA on the average pupil size and relative pupil size with the factors group, luminance equalization, emotional expression, and pupil size condition, only showed a main effect of group (for all statistical outcomes of the ANOVA, see Table 3; for pupil traces per observer, see Figure S3 in the Supplemental Material available online). However, a significant interaction effect was found between the factors luminance equalization and pupil-size condition (all other interactions were not significant; *p* > 0.05). Post-hoc t-tests indicated that only the in-group stimuli evoked a pattern in line with pupil mimicry (Table 4). A linear mixed-effects model showed similar results, with a significant interaction between observed pupil size, luminance equalization, and time (*F* = 28.425, *p* < 0.001; for all statistical outcomes of the model, see Table S2).

**Table 3:** Four-way ANOVA on observer’s average pupil size with all main-effects and relevant interactions between the factors group, luminance correction, emotion, and pupil size condition in experiment 2.

Factor	*F*-value	*p*-value	BF

Pupil size	0.97	0.383	0.012
Group	12.68	<0.001	>100
Luminance eq.	0.01	0.921	0.056
Emotion	7.99	0.006	2.114
Pupil size * Luminance eq.	21.58	<0.001	14.004

**Table 4:** Means, confidence intervals, and post-hoc t-test comparisons across pupil size conditions per equalization and group condition in experiment 2.

Condition	Compared conditions		Mean difference	Confidence interval	*t*-value	*p*-value	Cohen’s D	BF

Not eq./In-group	Sma.	Ori.	–0.15	–0.25/–0.05	2.89	**0.005**	0.402	665
	Ori.	Lar.	–0.04	–0.14/–0.06	0.76	0.453	0.098	
	Sma.	Lar.	–0.18	–0.26/–0.10	4.50	**<0.001**	0.554	
Equalized/In-group	Sma.	Ori.	0.05	–0.05/0.15	1.05	0.297	0.141	0.197
	Ori.	Lar.	–0.01	–0.12/0.10	0.16	0.872	0.024	
	Sma.	Lar.	0.04	–0.05/0.14	0.89	0.379	0.109	
Not eq./Out-group	Sma.	Ori.	–0.04	–0.14/0.05	0.94	0.352	0.128	0.259
	Ori.	Lar.	–0.01	–0.12/0.10	0.24	0.812	0.036	
	Sma.	Lar.	–0.06	–0.16/0.04	1.17	0.247	0.144	
Equalized/Out-group	Sma.	Ori.	0.04	–0.05/0.15	0.89	0.378	0.124	0.717
	Ori.	Lar.	0.05	–0.05/0.15	1.02	0.309	0.137	
	Sma.	Lar.	0.10	–0.01/0.20	1.89	0.063	0.232	

To summarize the results of experiment 2, the pupil mimicry effect was replicated for uncorrected in-group face stimuli, but when outgroup faces were presented or when the luminance of the pupil was equalized as a function of pupil size, no pattern in line with pupil mimicry effect was present.

## 4. Discussion

Previous studies have reported that observers’ pupils dilate when they see photos or videos of dilated pupils, compared to when they see photos or videos of constricted pupils: a phenomenon referred to as *pupil mimicry*. However, an alternative explanation, which we set out to investigate here, is that photos or videos of dilated pupils are simply darker than those of constricted pupils, and that this luminance difference drives the apparent pupil mimicry. Indeed, when removing differences in local brightness by strictly equalizing luminance values within the iris regions as a function of a changing pupil size, we observed no pattern of pupil mimicry. This means that it is likely that previous reports of pupil mimicry do not reflect a form of social mimicry, but rather a difference in luminance levels between stimuli.

The effect of luminance on the pupil contagion effect concurs with two previous findings. First, the observation that infant’s pupils only dilate in response to large pupils and do not constrict in response to small pupils (Fawcett et al., 2017) is explained by the non-linear, quadratic increase in luminance as function of the pupil’s surface size. The change in the pupil’s surface luminance from medium to small pupil size is smaller than the change in luminance from medium to large pupil size. In other words, it is not unexpected that only images with medium versus large pupil sizes result in significant differences in observers’ pupils, especially in noisy data sets such as those recorded in infant eye-tracking studies (Hessels, Andersson, Hooge, Nyström, & Kemner, 2015). Second, the pupil contagion effect is also strongest in medium to large pupils in Kret et al. (2015) and the effect sizes across pupil size conditions scale with the image surface covered by the pupils.

Surprisingly, we could only replicate pupil mimicry for in-group stimuli in which the pupil dynamically changed in size and the iris region was not corrected for luminance. We could not replicate pupil mimicry for stimuli with static pupil sizes or out-group stimuli with dynamic pupil sizes that were not equalized for luminance. The latter finding is not in line with previous studies that found pupil-mimicry effects for these conditions (Fawcett et al., 2017; Fawcett et al., 2016; Harrison et al., 2009; Harrison et al., 2006; Kret et al., 2015).

It is yet unknown what explains this discrepancy and we can only speculate what explains the null results. The most likely explanation is that the pupils in the current study were not conspicuous enough to evoke pupil mimicry. In line with the finding that observers tend to be unaware of pupil size (Naber, Stoll, et al., 2013) and together with the knowledge that the visibility of behavior is crucial for mimicry (Naber, Vaziri Pashkam, et al., 2013), observers may just not have processed the pupils sufficiently for action coupling to occur. Indeed, only 13 of the 60 observers in the experiment with static pupils indicated during debriefing that they had noticed the differences in pupil size across stimuli, while 64 out of 66 observers in the second experiment with dynamic pupils confirmed to have seen the pupils change in size. Although subliminal processing of pupil size outside awareness may still affect how emotional expressions are perceived (Harrison, Wilson, & Critchley, 2007) and how faces of individuals are trusted (Kret et al., 2015), this process may be too weak to cause pupil mimicry when the pupils are not salient enough or when the faces are less interesting because they belong to an unfamiliar group (i.e., Japanese).

Then why did previous studies find a pupil-mimicry effect? Harrison et al. (2006) used face stimuli that did not differ much from the current stimuli and still found pupil mimicry, although only for faces with a sad expression. They also found that the pupil-mimicry effect was associated with brain activity in regions implicated in social cognition and pupillary control. It is tempting to speculate that another, unknown factor related to the design or instructions in this study draw special attention to the pupils. In fact, Kret et al. (Kret & De Dreu, 2017; 2015; 2014) used slit stimuli in which the pupil suddenly increased or decreased in size per trial, just like the stimuli in experiment 2 of the current study. The sudden change in pupil size automatically draws the observer’s attention as motion is a highly conspicuous feature (Mahapatra, Winkler, & Yen, 2008), and attentional capture by moving bright or dark stimuli is known to affect pupil size (Mathôt, Dalmaijer, Grainger, & Van der Stigchel, 2014). These sudden changes were not present in the static stimuli in experiment 1. The absence of conspicuous motion signals and less (covert) attention for the eyes of static images may explain why we could not replicate pupil mimicry in this part of the study. Furthermore, although several features of the eyes were changed to improve the natural appearance of the eyes in Kret et al. (2015), these changes also made the pupil’s pop out more than normal. While such manipulation may not have been beneficial for the naturalness of the stimuli, it is not unlikely that such conspicuity manipulations are decisive factors for whether or not pupils stand out and are noticed enough to be mimicked.

The conspicuity of the pupils may have been affected by the luminance manipulations in the current study. The contrast in the eye regions was relatively lower for dilated pupils as compared to constricted pupils. The dilated pupils were increased in luminance towards the brightness of the surrounding iris, while constricted pupils were decreased in luminance, therewith in stronger contrast with the iris’ luminance. However, if contrast was a crucial factor for pupil mimicry, we should have observed a different pattern in the results of the luminance-equalized conditions, with significant rather than insignificant differences in pupil responses to eyes with static/original pupils versus constricting/small pupils.

As described in the introduction, conspicuous signals draw covert and overt attention (Itti & Koch, 2000) and may boost pupil mimicry. The effect of attention potentially explains the absence of pupil mimicry with the out-group persons: out-group individuals draw less attention, therewith suppressing pupil responses to facial content (Binda et al., 2013a; Mathôt et al., 2013; Naber, Alvarez, et al., 2013). We do want to denote that this proposition is mere speculation. An alternative explanation would be that the unfamiliarity with Japanese faces leads to more holistic processing, which consequentially removes a predominant focus on just the eyes. On the other hand, increased interest in the faces evokes a strong dilatory arousal response that may wash out more subtle pupil mimicry responses. The latter two interpretations are indeed in line with the finding that Japanese faces evoked stronger pupil dilation responses, but more research is needed to investigate which of the interpretations is most likely.

Note that overt fixation patterns may still be similar across in-group and out-group stimuli but it remains unknown whether the fixated information receives enough attention for accurate processing. Future work on this matter should therefore also take into account the conspicuity of the stimuli and pupils across conditions. Nonetheless, if pupil mimicry is a general, robust, and ecologically valid phenomenon that also manifests outside the lab in daily life in which the pupils of partners are not necessarily conspicuous, the effect should have been found in the current experiment with static stimuli as well.

A noteworthy finding of the current study was that faces with negative emotional expressions evoked more pupil dilation than positive and neutral expressions. This finding concurs with previous studies on pupil responses to emotional expressions and visual stimuli containing content with varying valences (Bradley, Miccoli, Escrig, & Lang, 2008; Burkhouse et al., 2017; Burkhouse, Siegle, Woody, Kudinova, & Gibb, 2015; Franzen, Buysse, Dahl, Thompson, & Siegle, 2009; Geangu, Hauf, Bhardwaj, & Bentz, 2011; Laeng et al., 2013; Silk et al., 2007; Reuten, van Dam, & Naber, 2018) (but see Bayer, Sommer, & Schacht, 2011; Nuske, Vivanti, & Dissanayake, 2016; Sepeta et al., 2012). The most probable explanation is that negative emotions evoke more sympathetic arousal, leading to activation of the dilator muscle of the pupil (Loewenfeld & Lowenstein, 1993). Negative emotions are encountered less often in real life and could therefore occur as more surprising to observers, a mechanism that is known to dilate the pupil (Lavín, San Martín, & Rosales Jubal, 2014; Preuschoff, t Hart, & Einhäuser, 2011; Satterthwaite et al., 2007).

In summary, the most important implication of the current study is the following: to ensure a confound-free design of stimuli, low-level image features such as luminance should be taken into account in when conducting pupillometry studies (Naber, Hilger, & Einhäuser, 2012). Especially when a relevant aspect of a stimulus (such as the eye’s pupils in faces) is manipulated in the experimental design, its low-level features need to be equalized across conditions (Willenbockel et al., 2010), even when this aspect covers only a small, local area of the stimulus.

Now it is evident that an observed increase in pupil size goes hand in hand with the decreased visibility of a brighter iris and increased visibility of a darker pupil, and that consequentially an observers’ pupils adapt to these changes in perceived brightness, it is tempting to question whether this effect should be called pupil mimicry at all. Although recently challenged (Naber, Vaziri Pashkam, et al., 2013), behavioral mimicry is typically interpreted as a social phenomenon (Chartrand & Bargh, 1999; Dijksterhuis & Bargh, 2001; Lakin et al., 2003). There is, however, nothing social about brightness perception. We do not exclude the possibility that increased attention for others’ pupils may still trigger or reflect social processes – fMRI studies indicate activation of brain areas associated with social cognition during pupil mimicry (e.g., Harrison et al., 2006) – but the current study provides evidence that pupil mimicry is not *caused* by a social mechanism.

## Additional Files

The additional file for this article can be found as follows:

10.5334/joc.34.s1 Supplementary Materials. Supplementary Figure S1–3 and Supplementary Table S1–2.

## Data Availability

Data can be accessed at osf.io/3jwvr.
