# Supplementary material for: Pupil Mimicry is the Result of Brightness Perception of the Iris and Pupil
Source: J Cogn. 2018 Jun 19;1(1):32. doi: 10.5334/joc.34 (PMC6634366; doi:10.5334/joc.34)
Supplement: Supplementary Materials. — Supplementary Figure S1–3 and Supplementary Table S1–2. [file joc-1-1-34-s1.pdf]

Supplementary Materials

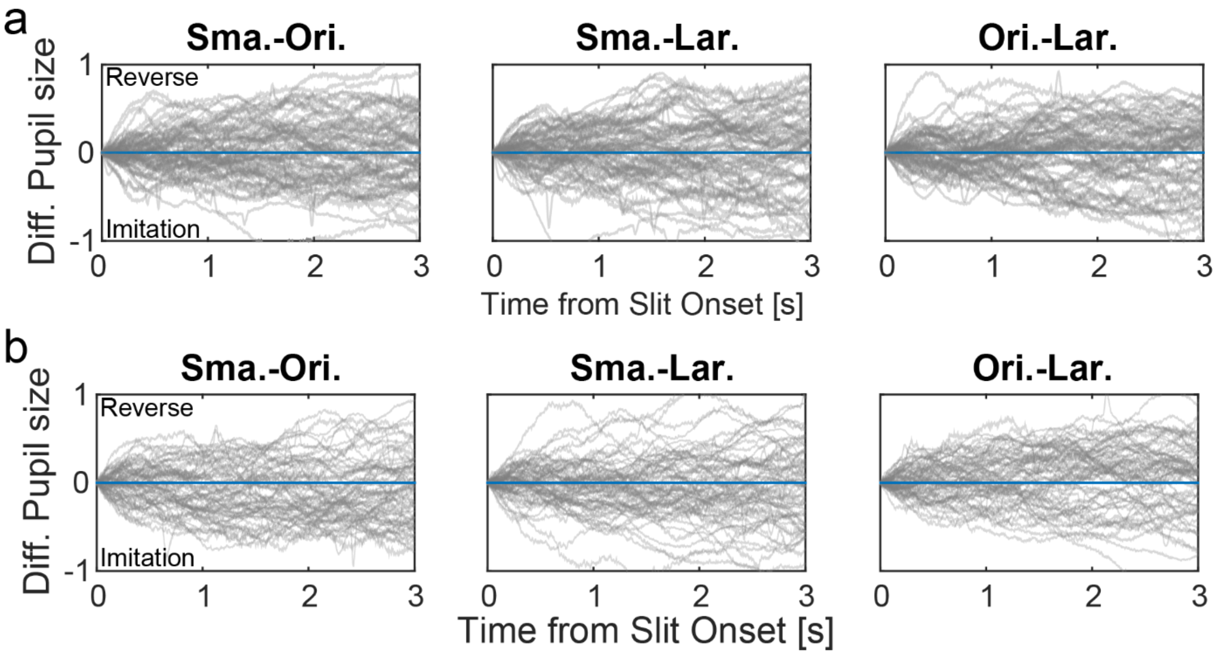

Figure S1. Difference in pupil size (z-value) as a function of time from onset of not equalized (a) or equalized (b) slit stimuli for small pupil minus original pupil size (left column), small minus large pupil size (middle column), and original minus large size (right column). The individual gray lines indicate the average difference per observer. Lines with negative values are in line with the pupil mimicry effect and lines with positive values indicate a reversed pupil mimicry effect.

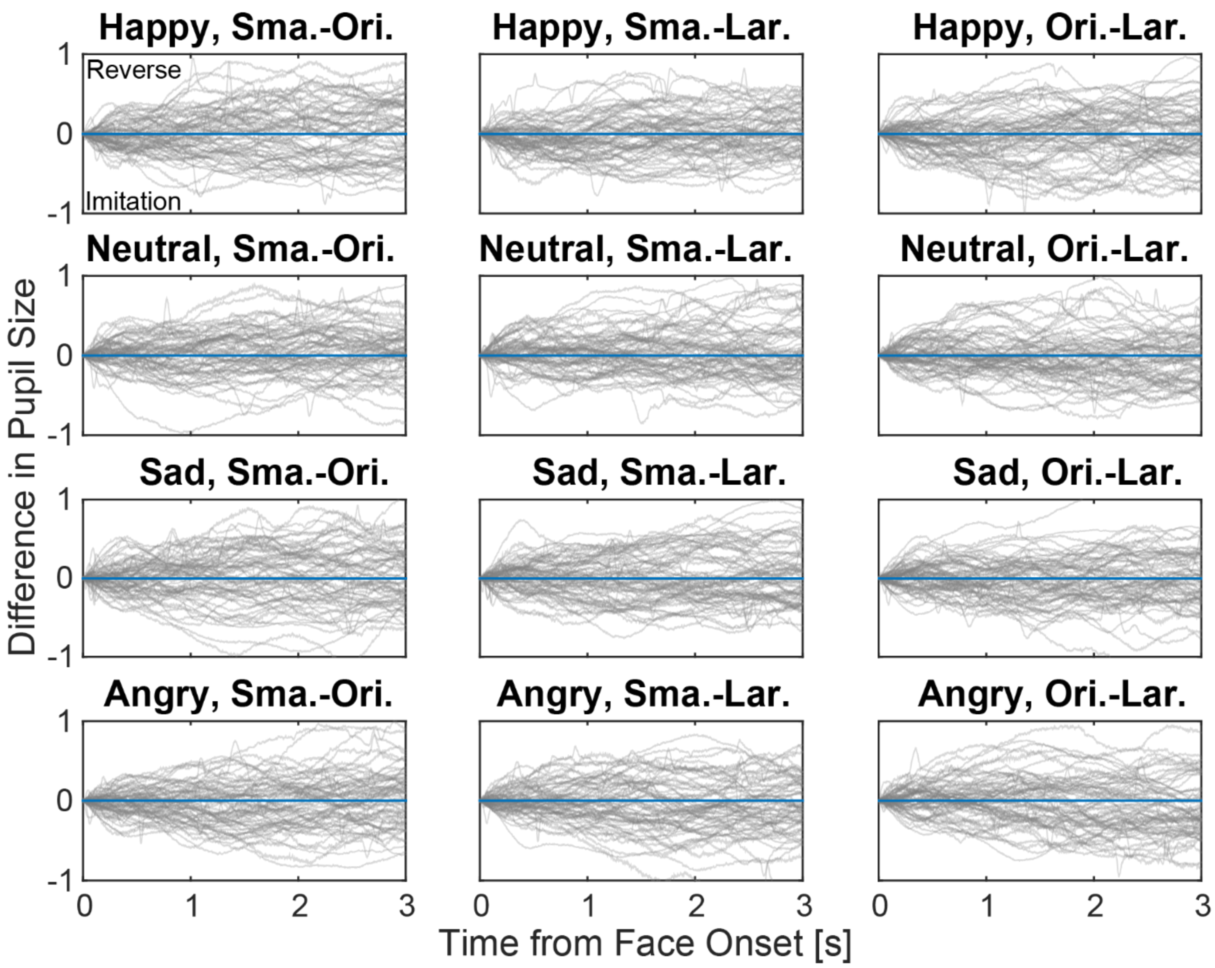

Figure S2. Same as Figure S1 but now for each observers' pupil responses to stimuli displaying full face images per emotional expression condition (rows).

Table S1.

Means, confidence intervals, and post-hoc t-test comparisons of average pupil size between pairs of emotion conditions of block 3 in experiment 1.

Compared emotions		Mean difference	Confidence interval	*t*-value	*p*-value	*Cohen's d*	*BF*
Happy	Neutral	-0.015	-0.06/0.03	0.69	0.490	0.059	0.178
	Sad	-0.075	-0.13/-0.03	3.01	**0.004**	**0.267**	**8.171**
	Angry	-0.097	-0.15/-0.05	4.01	**<0.001**	**0.350**	**132.733**
Neutral	Sad	-0.061	-0.11/-0.01	2.40	**0.019**	**0.215**	**1.982**
	Angry	-0.082	-0.12/-0.04	4.05	**<0.001**	**0.297**	**150.299**
Sad	Angry	-0.022	-0.07/0.02	0.89	0.379	0.070	0.205

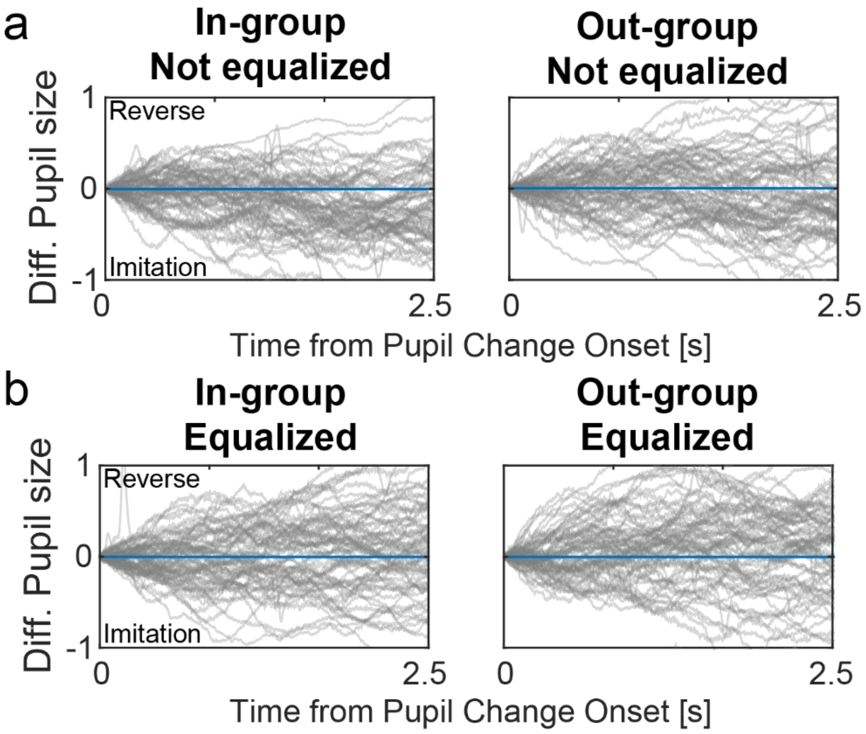

Figure S3. Same as Figure S1-S2 but now for each observers' pupil responses to stimuli displaying videos of slits of the eyes of in-group (left) or out-group persons (right) with dynamic pupil sizes that were either not equalized (a) or equalized for luminance (b) as a function of pupil size.

Table S2.

Results from a linear mixed-effects model with the factors time, observed pupil size, and luminance equalization.

**Fixed Effect**	***F***	***p***
Intercept	0.006	.941
Time (linear trend)	25.371	**< 0.001**
Observed pupil size	< 0.001	.992
Luminance equalization	0.067	.796
Time × Observed pupil size	2.886	.089
Time × Luminance equalization	2.818	.093
Observed pupil size × Luminance equalization	0.071	.790
Time × Observed pupil size × Luminance equalization	28.425	**< .001**
